# Prostate Cancer Incidence and Mortality in Barbados, West Indies

**DOI:** 10.1155/2011/565230

**Published:** 2011-04-17

**Authors:** Anselm J. M. Hennis, Ian R. Hambleton, Suh-Yuh Wu, Desiree H.-A. Skeete, Barbara Nemesure, M. Cristina Leske

**Affiliations:** ^1^Chronic Disease Research Centre, The University of the West Indies, Jemmott's Lane, St. Michael, Barbados; ^2^Department of Preventive Medicine, Stony Brook University Medical Center, Stony Brook, NY 11794-8036, USA; ^3^Ministry of Health, Jemmott's Lane, Bridgetown, Barbados; ^4^Faculty of Medical Sciences, The University of the West Indies, St. Michael, Barbados

## Abstract

We describe prostate cancer incidence and mortality in Barbados, West Indies. We ascertained all histologically confirmed cases of prostate cancer during the period July 2002 to December 2008 and reviewed each death registration citing prostate cancer over a 14-year period commencing January 1995. There were 1101 new cases for an incidence rate of 160.4 (95% Confidence Interval: 151.0–170.2) per 100,000 standardized to the US population. Comparable rates in African-American and White American men were 248.2 (95% CI: 246.0–250.5) and 158.0 (95% CI: 157.5–158.6) per 100,000, respectively. Prostate cancer mortality rates in Barbados ranged from 63.2 to 101.6 per 100,000, compared to 51.1 to 78.8 per 100,000 among African Americans. Prostate cancer risks are lower in Caribbean-origin populations than previously believed, while mortality rates appeared to be higher than reported in African-American men. Studies in Caribbean populations may assist understanding of disparities among African-origin populations with shared heredity.

## 1. Introduction

Prostate cancer rates are higher in westernized African-origin populations when compared to other ethnic and racial groups [1, 2]. In the United States, prostate cancer remains the principal malignancy in African-American men and the leading cause of cancer-related death in this group [3, 4]. Similar findings have been reported from the English-speaking Caribbean [5], where the majority of the population shares a common heredity with African Americans, as a result of the West African diaspora [6]. Despite the importance of prostate cancer as a cause of ill health and mortality in the Caribbean, there remains limited information about disease rates, risk factors, and the clinical and public health implications for the region [7–10]. 

The Barbados National Cancer Study (BNCS), funded by the National Institutes of Health, was established in 2002 to document the incidence and risk factors for prostate and breast cancer, among the country's men and women, respectively. Such malignancies are known to be the most frequent among the island's residents [11]. The African-descent Barbados population shares a common heredity with African Americans (but with lower admixture) [12], as well as high rates of lifestyle-related noncommunicable disease [13–16]. While investigation of conditions relevant to African Americans in the US may be confounded by associations with socioeconomic factors, the public care system in Barbados provides easy access to comprehensive healthcare at no cost, thus minimizing such concerns. As demonstrated in our previous work, findings from the Barbadian population may therefore have direct relevance to the health of African-American populations [17]. 

In addition to collecting data on prostate cancer incidence, a key outcome of the BNCS, we also utilized the established research infrastructure to collect additional information on prostate cancer-related mortality. The purpose of this paper is to present the first comprehensive data about prostate cancer incidence and mortality in Barbados, West Indies, and provide comparisons with African Americans. Although African Americans are consistently reported to have the highest prostate cancer rates globally, data from Jamaica [8] suggested that Caribbean men might even have higher disease rates. This key clinical and public health concern, underpinned the conduct of this study.

## 2. Materials and Methods

All histologically confirmed cases of prostate cancer were ascertained from records held at the Pathology Department of the Queen Elizabeth Hospital, Bridgetown, the sole public tertiary care institution on the island, where all pathological specimens are evaluated. Other data sources such as patient charts and pathology reports were also reviewed, and cases of recurrent prostate cancer or disease occurring in non-residents (domiciled for less than 6 months a year) were excluded. Incidence estimates presented here were based on data collected by the BNCS between 2002 and 2008. 

To ascertain prostate cancer-related mortality, we reviewed all death certificates for the period January 1, 1995 to December 31, 2008, selecting certificates that listed a diagnosis of prostate cancer alone or in conjunction with other causes of death. Unlike the requirements of the US Standard Certificate of Death, death registration in Barbados during this period did not require physicians to record underlying cause of death.

To address potential overcounting of deaths attributed to prostate cancer [18], we developed a nosological algorithm based on independent review by two clinicians to determine if prostate cancer was (i) unlikely to be the cause of death, (ii) the probable cause (multiple causes of death cited, death likely attributable to metastatic prostate cancer), or (iii) the definite cause (prostate cancer being the single listed cause of death; death attributable to metastatic prostate cancer with other listed conditions unlikely to have directly caused death). Two clinicians (A. J. M. Hennis and D. H.-A. Skeete) resolved any diagnostic discrepancies by consensus. 

Informed consent was obtained from all BNCS participants, and the study protocols conformed to the Declaration of Helsinki. In addition, the current study was conducted as a clinical audit of a national dataset on behalf of the Ministry of Health. The data were delinked such that participants could not be identified. This study was approved by the University of the West Indies/Ministry of Health Institutional Review Board. 

### 2.1. Statistical Methods

To calculate crude prostate cancer incidence rates per 100,000 years of observation, we divided the number of incident cases by the number of males (all ages) in the Barbados population and multiplied the results by 100,000. Age-specific incidence rates were calculated in 18 age groups (stratified by 5-year increments as follows: 0–4 years, 5–9 years, 10–14 years, through 85 years, and older). We derived age-standardized rates (with 95% confidence intervals), using the direct method, to allow age-independent comparisons with other studies. These rates were estimated based on three standard populations: the 2000 US standard population [19] and the IARC European and World standard million populations [20]. We also compared age-standardized prostate cancer incidence in Barbados (based on the 2000 US standard population) with US rates (Surveillance Epidemiology and End Results (SEER)) [21], for the period 2002 to 2007, and calculated age-stratified and age-standardized death rates for the period 1995 to 2008. Direct comparisons of prostate cancer incidence in Barbados and the US were made utilizing incidence rate ratios derived from log-linear models, according to age and year of diagnosis. Exact Poisson confidence intervals were calculated for crude and age-stratified rates. Given the utility of the Gamma approximation in providing more accurate estimates with small numbers, we used this method to estimate age-standardized confidence intervals [22]. 

We used previously described approaches to calculate age-stratified and age-standardized death rates in Barbados for the 14-year period 1995–2008. Because of uncertainty about prostate cancer as an underlying cause of death, we present two mortality rates, based on (i) deaths restricted to those definitely attributed to prostate cancer and (ii) including all deaths, definitely or probably attributed to prostate cancer; deaths unlikely due to prostate cancer were excluded. US cancer mortality information is provided as rates only (without documentation of actual numbers), and stratified by age groups <65 and 65 years and older. Our comparisons with US mortality data (presented in Table 5), are therefore limited by the unavailability of detailed US data. All analyses were carried out using Stata (Version 11, StataCorp LP, College Station, Texas, USA).

## 3. Results

During the initial six and a half year study period (July 1, 2002–December 31, 2008) of the BNCS, 1,101 men were diagnosed with histologically confirmed prostate cancer.  Table 1 presents age-specific prostate cancer incidence in Barbados. Age at presentation ranged from 25 to 99 years, with a median age of 68 years (interquartile range: 61 to 74 years). Prostate cancer incidence increased from 6.0 (95% confidence interval: 1.6–15.3) per 100,000 at ages 40 to 44 years, to a peak of 1,026.6 (95% CI: 898.8–1,167.6) per 100,000 in men aged 70 to 74 years, and declined thereafter. The overall crude incidence rate was 131.0 (95% CI: 123.4–139.0) per 100,000, with rates of 160.4 (95% CI: 151.0–170.2), 163.1 (95% CI: 153.4–173.3), and 112.0 (95% CI: 105.2–119.3) per 100,000 standardized to the US, European, and World populations, respectively. Comparable rates in the US varied according to race, such that overall prostate cancer incidence in African-American and White men was 248.2 (95% CI: 246.0–250.5) and 158.0 (95% CI: 157.5–158.6) per 100,000, respectively. 

**Figure 1:**
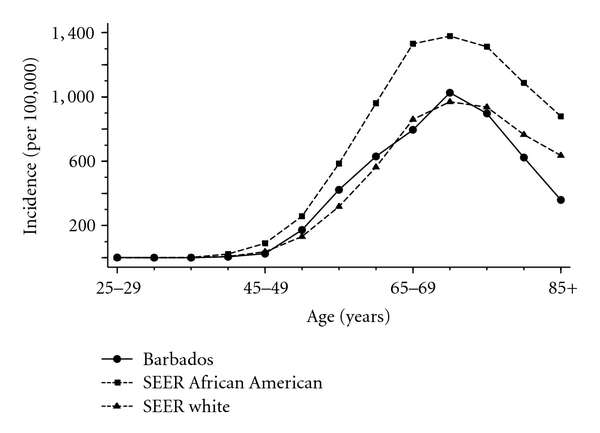
Age-specific incidence of prostate cancer in Barbados (2002–2008) and the US SEER 2000–2007 [21]. Note: SEER incidence rates based on malignant and in situ disease.

Figure 1 presents age-specific prostate cancer incidence in Barbadian men and comparable data from the US. Men aged 40–44 years comprised the youngest group which developed prostate cancer and rates were approximately fourfold higher in African Americans (22.9 (95% CI: 21.1–24.9)) than Barbadians (6.0 (95% CI: 1.6–15.3); log linear model comparison, *P* = .01). 

The highest prostate cancer incidence among both Barbadians and African Americans occurred among men aged 70–74 years: 1,026.6 (95% CI: 898.8–1,167.6) and 1,378.6 (95% CI: 1,347.4–1,410.3) per 100,000, respectively, still being comparatively higher in the latter group (risk ratio: 1.34, 95% 1.18–1.53, *P* < .001).

Table 2 presents secular trends in incident prostate cancer during the period 2002 to 2008. In contrast to an age-standardized rate of 158.8 (95% CI: 148.6–169.5) per 100,000 among Barbadian men, prostate cancer incidence rates among African Americans always exceeded 225 per 100,000 between 2002 and 2007. 

Table 3 presents data on age-specific and age-standardized prostate cancer incidence in Barbadian and African-American men. Incidence rate ratios (IRR) confirmed lower rates of newly diagnosed prostate cancer in Barbadians. Rates among African Americans were approximately 1.5 times higher (among men aged 50 to 79) and in excess of 3.5 times higher among the youngest men (aged 40 to 44 years) (*P* < .001 in all age group comparisons from 45 years and older). Analysis of secular trends in disease incidence during the 6-year period 2002 to 2007 confirmed overall prostate cancer incidence to be between 1.3 and 1.9 times higher in African-American men (*P* < .001 in all annual comparisons).

Table 4 presents age-stratified and age-standardized prostate cancer death rates in Barbados between 1995 and 2008, per 100,000 person-years of observation. There were a total of 1,496 death certifications citing prostate cancer as a cause of death. Of these, 943 (63.0%) cited prostate cancer as the only cause of death, and 553 (37.0%) cited one or more other causes. These 553 certifications with multiple causes of death were reviewed by two clinicians, and prostate cancer was determined to either be the probable cause of death (294, 19.7% of all deaths) or not likely to be the cause of death (259, 17.3% of all deaths). 

The number of deaths from prostate cancer progressively increased with age, with the distribution of definite prostate cancer mortality by 10-year age groups being 1 or 0.1% (aged 39 or less), 7 or 0.7% (aged 40–49), 25 or 2.6% (aged 50–54), 25 or 2.6% (aged 55–59), 61 or 6.5% (aged 60–64), 80 or 8.5% (aged 65–69), 132 or 14% (aged 70–74), 173 or 18.3% (80–84), 225 or 24% (aged 85+). Comparable combined probable and definite prostate cancer mortality by 10-year age groups were 1 or 0.1% (aged 39 or less), 7 or 0.6% (40–49), 54 or 4.4% (50–59), 175 or 14.1% (60–69), 396 or 32.0% (70–79), 602 or 48.7% (80 years and older). The crude death rate for definite prostate cancer deaths was 48.6 (95% CI: 45.5–51.8) per 100,000 and for definite and probable prostate cancer deaths, 63.7 (95% CI: 60.2–67.3) per 100,000. 

The equivalent age-standardized rates (US standard 2000 population) were 62.7 (95% CI: 58.8–66.9) deaths per 100,000 person-years for definite prostate cancer deaths and 82.7 (95% CI: 78.1–87.4) per 100,000 for combined definite and probable prostate cancer deaths.

**Figure 2:**
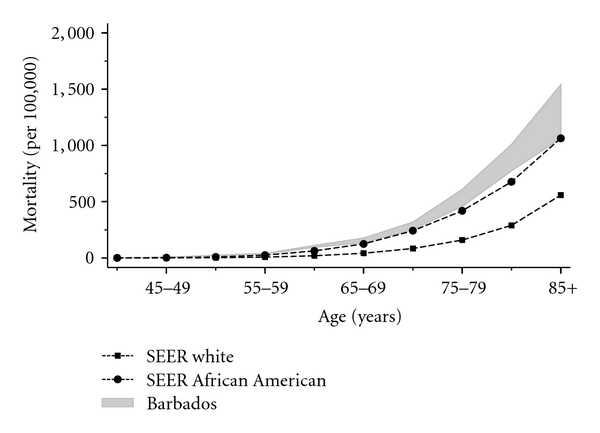
Age-specific death rates of prostate cancer in Barbados (1995-2009) (The band representing the distribution of age-specific mortality is composed of “definite” (lower limit) and “definite and probable” (upper limit) prostate cancer mortality. ) and the US SEER 2002–2006.

Figure 2 and Table 5 present data comparing mortality from prostate cancer in the Barbadian and US populations between 1995 and 2008. In Barbados, overall prostate cancer mortality (probable and definite) ranged from 63.2 to 101.6 per 100,000, while overall rates among African Americans ranged from 51.1 to 78.8 per 100,000. During the period 1995 to 1998, overall rates of “definite” prostate cancer mortality in Barbados were lower than reported in African Americans (52.3–67.7 and 72.8–78.3 per 100,000, resp.). In contrast, mortality rates in Barbados due to definite prostate cancer between 1999 and 2006 (barring the year 2001) were consistently higher than those reported in African Americans, who demonstrated a trend to declining related mortality. With regards to age-stratified, Barbadian men aged less than 65 years had higher mortality attributed to definite prostate cancer than African Americans (except for 1998 and 2003). Among men aged 65 years and older, death rates attributable to probable and definite prostate cancer were higher among older Barbadian men between 1998 and 2006. Figure 2 compares age-specific prostate cancer mortality, rates in White and African Americans, with Barbadians. Based on “definite” prostate cancer-related deaths, age-specific mortality rates in Barbados were at least as high as those in African Americans for all age groups and higher when prostate cancer was considered the combined “definite and probable” cause of death. Mortality increased with older age in each of the three ethnic groups.

## 4. Discussion

A total of 1101 incident prostate cancer cases were recorded in Barbados during the six and a half year study period (July 2002 to December 2008), for an incidence rate of 160.4 per 100,000 (standardized to the US population). This prostate cancer incidence was similar in Barbadian and White American men, in contrast to rates in African-American men, which were about one and a half times higher. The age-specific pattern of incident disease was similar in both African-descent groups, rising to a peak by ages 70 to 74 years, and declining thereafter. Younger African-American men aged 40 to 44 years, however, experienced a nearly fourfold higher occurrence of prostate cancer than similarly aged Barbadian men (groups based on relatively low numbers). While overall prostate cancer mortality rates demonstrated a clear decline in African Americans during the period from 2000 to 2006, they were higher in Barbadian men. 

Prostate cancer ranks as the sixth most frequent incident tumor worldwide, accounting for nearly 10% of all cancers in men [23]. Prevalence is higher in developed than developing countries, where 15% and 4% of men are affected, respectively. 

There are clear associations between prostate cancer occurrence and race (or ethnicity) such that African-American men experience the highest rates globally, Caucasian men in North America and Europe, high and intermediate rates, while Asian men have relatively low disease rates [1, 23, 24]. Based on comprehensive global population-based cancer data reported continuously over a 20-year period by the International Agency for Research in Cancer (IARC), the highest prostate cancer incidence and mortality worldwide was documented in African-American men [1]. Incidence and mortality rates in African-American men were approximately sixty times and twelve times, respectively, those recorded in Chinese men (known to be at lowest risk). 

Few data exist on prostate cancer rates in West African and Caribbean populations who share a common heredity with African-American populations. Much of the currently available data have been reported from the Globocan database, often the only source of cancer data from many regions of the world [5]. It is important to note that the authors caution that the “degree of detail and quality of the data vary considerably” and provide the caveat that the quality of information presented depends “on the extent and accuracy of locally available data.” While there are extensive data on prostate cancer rates among African-American populations, few data are available for West African populations, who are thought to have relatively high disease rates [25].

Prostate cancer is listed as the second most frequent malignancy among West African men with rates of approximately 19 per 100,000 [26]. Two independent hospital-based studies conducted in Nigeria reported prostate cancer incidence rates of 61.3 per 100,000 and 127 per 100,000 [27, 28]. However, a critical reappraisal by Ben-Shlomo et al. highlighted major errors in these estimates [29]. Their revised calculations reduced these estimated incident rates to 6.1 per 100,000 and 21.1 per 100,000, respectively. Ben-Shlomo et al. similarly recalculated prostate cancer incidence reported in a hospital-based case series conducted in Cameroon at 0.2 per 100,000 [29], rather than the reported rate of 93.8 per 100,000 [30]. These hospital-based studies conducted in the 1980s to mid 1990s would have led to erroneous estimates of cancer incidence and reevaluation of these data provides evidence for relatively low prostate cancer rates in West Africa. 

Further evidence of relatively low prostate cancer incidence rates in West Africa comes from cancer registry data from Guinea (8.1 per 100,000), Mali (6.3 per 100,000), and Gambia (1.2 per 100,000) a decade and a half ago [31, 32]. 

Information is now available from studies of first generation Black Caribbean and African men resident in the United Kingdom. Risk of incident prostate cancer was three times higher in African Caribbean compared to European men resident in North East London [33]. The larger Prostate Cancer in Ethnic Subgroups (PROCESS) study of incident prostate cancer conducted in London and Bristol over a longer (5-year) period documented adjusted rates (based on the European standard population) of 173.1 per 100,000 for Black Caribbean men and 139.3 per 100,000 for Black African men, compared to 56.4 per 100,000 in White British men [29]. Standardized to the US population, prostate cancer incidence was 166 per 100,000 among Black British men, inline with the rate of 158.9 per 100,000 among Barbadian men. 

### 4.1. Prostate Cancer Incidence in the Caribbean

The comparatively lower rates of prostate cancer in Barbadian men contrasts with earlier reports from Jamaica, West Indies, where the reported average incidence rate of 304 per 100,000 (1989 to 1994) exceeded comparable rates in African-American men (249 per 100,000) [8]. The accuracy of the Jamaican rates has also been challenged by Ben-Shlomo et al., who estimated the corrected prostate cancer incidence (unadjusted) to be less than one-fourth the original rate at 70 per 100,000 [29]. They suggested that standardization of these rates to the US population would reduce this estimate even further, given the comparably younger average age of the Jamaican population. Based on data from the largely urban-based Jamaica Cancer Registry, Hanchard et al. [7] and Gibson et al. [10] reported standardized prostate cancer incidence rates (world population) of 56.4 and 65.6 per 100,000, respectively, over consecutive 4-year periods, (1993 to 1997 and 1998 to 2002). These rates are entirely consistent with those estimated by Ben-Shlomo et al. [29]. 

Limited data on prostate cancer incidence are available from the French Caribbean islands, which also have predominantly West African-origin populations. There has been an active cancer registry in Martinique since 1983, and Dieye et al. [34] reported a cumulative age-standardized (world population) prostate cancer incidence of 80.8 per 100,000 (between 1981 and 2000). Annual rates increased over time to 161 per 100,000 in 2000, comparable to rates in Barbados. Prostate cancer incidence was also similar in Guadeloupe at a rate of 168 per 100,000 [35]. Based on registry reports, prostate cancer rates (standardized to the world population) were relatively lower in Cuba (Hispanic Caribbean: 34.9 per 100,000 in 1999) [36].

In sum, prostate cancer incidence is similar in Black Caribbean populations resident in the United Kingdom and French and Barbadian populations resident in the Caribbean, and is around the order of 160 per 100,000. This is in sharp contrast to earlier reported incidence rates of around 300 per 100,000 from Jamaica, which appear to be an overestimate. Our findings, supported by other reports, therefore indicate that the impression of the Caribbean being a high-risk region for prostate cancer is incorrect. 

The impact of screening must be specifically addressed, as it significantly influences prostate cancer incidence. Increased utilization of prostate-specific antigen (PSA) screening and higher uptake of transurethral prostatic resection significantly contributed to increased prostate cancer rates in the US [37–39]. Anecdotally, PSA screening in the Caribbean region still lags behind uptake in more developed regions. Bunker et al. reported a prevalence of 10% for screen-detected prostate cancer in Tobago, West Indies [9], which highlights the potential impact of increased utilization of screening on disease rates.

### 4.2. Prostate Cancer Mortality

Cancer is now the second leading cause of death in the US accounting for 1 in 4 deaths [40, 41]. Racial disparities in cancer mortality persist in the US, although survival has improved in virtually all ethnic groups [3]. As such, survival after a cancer diagnosis still remains poorer among Black than White Americans [42]. Prostate cancer mortality has been approximately two times higher among Black than White Americans in recent decades [43], and current global comparisons confirm worse mortality outcomes in African-American men [24]. Vital statistics from the UK indicate that prostate cancer mortality rates among men born in West Africa and the West Indies are two to three times higher than overall rates in the male population [2]. Limited data also confirm lower 5-year survival among Black than White men following a prostate cancer diagnosis [44]. 

Factors expected to affect outcomes, such as preexisting comorbid conditions and access to care or uptake of PSA screening, did not emerge as independent predictors of ethnic disparities in prostate cancer mortality in a recent meta-analysis [45]. These findings must, however, be viewed with caution, given the substantial body of information to the contrary [46–48]. Tumor grade (biological aggressiveness) [49], underpinned by specific genetic differences [50], may partly explain racial disparities in prostate cancer mortality.

Mortality rates following a prostate cancer diagnosis in recent years are at least similar, in Barbadian and African-American men (and to some extent higher in the former group). This finding is a cause for concern, given the comparatively lower prostate cancer incidence in Barbados. 

A unique strength of this study concerns the access to all histologic samples analyzed at the island's sole pathology department, leading to near complete ascertainment of all incident prostate cancer cases. The strict criteria we used to establish a diagnosis of prostate cancer, which required histological confirmation, would have however reduced the true number of cases, as standard practice includes observation or empiric treatment of elderly men without proceeding to biopsy. This practice would have resulted in an underestimate of prostate cancer incidence in this study. While information about the uptake of biopsy in the diagnosis of prostate cancer nationally would have been useful, this was beyond the scope of this study. It is also our view that there is a lower uptake of PSA screening in Barbados compared to the US, which coupled with lower biopsy rates would have also led to an underestimate of prostate cancer incidence. The majority of the Barbadian population is of African descent, with fewer than 5% of men self-reporting White race (2000 household census). This low percentage of non-African descent Barbadians is likely to have minimal effect on our prostate cancer rates and allows comparison with other similar populations.

A potential limitation is that differences in death certification processes would have affected the comparability of prostate cancer mortality rates in Barbados versus the United States. We therefore developed an algorithm to classify prostate cancer-related deaths. This algorithm used an extremely conservative approach, with death only attributed to prostate cancer, where it was listed as the only cause, or the death certificates provided clear evidence of death due to metastatic prostate cancer. This methodology would have likely led to underestimates of prostate cancer as a “definite” cause of mortality and reduced related mortality rates accordingly. This research was based on comprehensive access to information that allowed identification, for the first time, of all incident prostate cancer cases and deaths occurring on the island, although the caveats noted would have to be considered in the interpretation of our findings.

## 5. Conclusions

This paper describes the incidence and mortality from prostate cancer in Barbados, West Indies, mainly populated by individuals of West African descent. Incidence rates were found to be consistent with those of similar populations in the Caribbean, as well as Caribbean populations in the United Kingdom, and lower than rates reported in African Americans. In spite of easy access to comprehensive health care, freely available in the public sector, prostate cancer mortality was comparatively higher than rates experienced by African-American populations. This observation calls for clinical and public health approaches to improve detection of disease, in order to reduce the high mortality rates currently experienced.

## Figures and Tables

**Table 1:** Age-specific and age-standardized incidence of prostate cancer in Barbados between July 01, 2002 and December 31, 2008, per 100,000 person-years of observation.

Age group (years)	Number of cases	Person-years*	Age-specific rate	95% CI
0–4	0	60,793	0.0	0–6.1
5–9	0	65,446	0.0	0–5.6
10–14	0	65,335	0.0	0–5.7
15–19	0	65,960	0.0	0–5.6
20–24	0	62,648	0.0	0–5.9
25–29	1	68,960	1.5	0–8.1
30–34	0	67,359	0.0	0–5.5
35–39	0	71,536	0.0	0–5.2
40–44	4	66,942	6.0	1.6–15.3
45–49	14	55,952	25.0	13.7–42.0
50–54	79	45,743	172.7	136.7–215.2
55–59	129	30,517	422.7	352.9–502.3
60–64	175	27,778	630.0	540.1–730.6
65–69	203	25,526	795.3	689.6–912.5
70–74	232	22,598	1,026.6	898.8–1,167.6
75–79	147	16,384	897.2	758.0–1,054.5
80–84	74	11,875	623.2	489.3–782.3
85+	33	9,183	359.4	247.4–504.8
Unknown age	10	—	—	
Crude rate	1,101	840,535	131.0	123.4–139.0
Age-standardized rates (US)	—	—	160.4	151.0–170.2
Age-standardized rates (Europe)	—	—	163.1	153.4–173.3
Age-standardized rates (World)	—	—	112.0	105.2–119.3

*85+ age group rounded up to 9,183 to maintain correct person-year total.

**Table 2:** Short-term secular change in prostate cancer incidence in Barbados and in African Americans.

Year	Follow-up months (days)	Cases	Person-yrs exposure	Age-standardized incidence rate (SEER US 2000 standard population)	
				Barbados	SEER (African Americans; 17 registries)
2002	6 (183)	78 (77)	64,766	146.3 (115.3–183.3)	273.1 (266.3–280.0)
2003	12 (365)	159 (155)	129,177	151.2 (128.3–177.2)	248.3 (241.9–254.8)
2004	12 (366)	195 (192)	129,530	184.1 (158.9–212.3)	245.2 (238.9–251.6)
2005	12 (365)	167 (166)	129,177	159.3 (135.9–185.7)	227.1 (220.9–233.4)
2006	12 (365)	135 (135)	129,177	127.3 (106.7–150.9)	225.8 (220.0–231.7)
2007	12 (365)	188 (187)	129,177	178.2 (153.5–205.9)	227.8 (222.1–233.7)
2008	12 (366)	179 (179)	129,530	169.3 (145.3–196.3)	*n*/a*
2002–2007	66 (2,009)	922 (912)	711,004	158.8 (148.6–169.5)	248.2 (246.0–250.5)
2002–2008	78 (2,375)	1,101 (1,091)	840,535	160.4 (151.0–170.2)	—

SEER rates based on malignant and in situ disease.

*SEER data for 2008 not available (*n*/*a*).

**Table 3:** Incidence rate ratios (IRRs) comparing age-specific incidence rates and secular incidence rates between Barbados and the United States (SEER 17 registries; African Americans) [6].

Characteristic	IRR	95% CI
Age^1^		
40–44	3.84	1.43–10.25
45–49	3.57	2.11–6.05
50–54	1.49	1.20–1.87
55–59	1.39	1.16–1.65
60–64	1.53	1.31–1.77
65–69	1.67	1.46–1.92
70–74	1.34	1.18–1.53
75–79	1.46	1.24–1.72
80–84	1.74	1.38–2.20
85+	2.45	1.73–3.46

Year^2^		
2002	1.87	1.50–2.34
2003	1.69	1.44–1.98
2004	1.34	1.16–1.55
2005	1.45	1.24–1.69
2006	1.77	1.50–2.11
2007	1.30	1.12–1.50

^1^Age-specific incidence rate ratios calculated for ages 40–44 and older—below age 40 there was just one case of prostate cancer in Barbados.

^2^Annual incidence rate ratios calculated for ages 40–44 and above using Poisson regression, adjusted for age. IRRs based on unadjusted *crude rates* are (2002) 1.35 (1.08–1.69), (2003) 1.26 (1.07–1.48), (2004) 1.01 (0.88–1.17), (2005) 1.12 (0.96–1.31), (2006) 1.43 (1.20–1.69), (2007) 1.06 (0.92–1.23).

**Table 4:** Age-stratified and age-standardized definite and probable death rates from prostate cancer in Barbados between Jan 01, 1995 and Dec 31, 2008, per 100,000 person-years of observation.

Age group (years)	No. of definite/(probable and definite) prostate cancer deaths	Person-years	Mortality Definite prostate cancer death (95% CI)	Mortality Definite and probable prostate cancer deaths (95% CI)
0–4	0	140,154	—	—
5–9	0	150,870	—	—
10–14	0	150,615	—	—
15–19	0	152,055	—	—
20–24	0	144,420	—	—
25–29	0	158,970	—	—
30–34	1	155,280	0.6 (0–3.6)	0.6 (0–3.6)
35–39	0	164,910	—	—
40–44	0/0	154,320	—	—
45–49	7/7	128,985	5.4 (2.2–11.2)	5.4 (2.2–11.2)
50–54	25/26	105,450	23.7 (15.3–35.0)	24.7 (16.1–36.1)
55–59	25/28	70,350	35.5 (23.0–52.5)	39.8 (26.4–57.5)
60–64	61/71	64,035	95.3 (72.9–122.4)	110.9 (86.6–139.9)
65–69	80/104	58,845	136.0 (107.8–169.2)	176.7 (144.4–214.1)
70–74	132/166	52,095	253.4 (212.0–300.5)	318.6 (272.0–371.0)
75–79	173/230	37,770	458.0 (392.3–531.6)	608.9 (532.8–692.9)
80–84	212/276	27,375	774.4 (673.7–866.0)	1,008.2 (892.8–1,134.5)
85+	225/326	21,165	1,063.1 (928.7–1,211.4)	1,540.3 (1,377.6–1,716.9)
Age unknown	2/2	—	—	
Total	943/1,237	1,937,664		
Crude rate			48.6 (45.5–51.8)	63.7 (60.2–67.3)
Age-standardized rates (US)	—	—	62.7 (58.8–66.9)	82.7 (78.1–87.4)
Age-standardized rates (Europe)	—	—	49.6 (46.4–53.0)	64.3 (60.7–68.2)
Age-standardized rates (World)	—	—	29.7 (27.7–31.9)	38.1 (35.8–40.6)

**Table 5:** Annual age-standardized definite and probable death rates from prostate cancer in Barbados compared to US SEER data (African Americans) between Jan 01, 1995 and Dec 31, 2009, per 100,000 person-years of observation.

	Prostate cancer death rates					
Year	Barbados^1^			SEER (African Americans)		
	All	<65 years	65+ years	All	<65 years	65+ years
1995	67.7/82.9	6.7/7.9	489.5/602.0	78.2	6.7	572.5
1996	52.3/63.2	7.6/8.6	361.2/440.4	78.8	6.5	579.2
1997	66.8/72.7	10.8/10.8	453.6/500.4	74.3	6.3	544.2
1998	61.4/74.8	4.2/4.2	457.1/562.9	72.8	6.3	532.4
1999	72.5/84.7	8.5/8.5	514.3/611.1	70.1	5.8	514.8
2000	70.5/86.2	9.5/10.6	491.9/609.4	68.7	5.4	505.8
2001	60.5/81.4	10.1/13.3	408.3/552.4	66.5	5.4	488.9
2002	78.7/88.8	10.8/10.8	548.4/628.2	63.0	5.4	461.2
2003	63.7/84.5	3.2/5.2	481.8/632.3	58.0	5.4	421.4
2004	63.4/85.4	6.2/7.4	458.7/625.4	56.2	5.0	410.4
2005	64.7/92.7	8.4/8.4	454.0/675.0	54.2	5.0	393.8
2006	53.2/75.4	11.5/13.7	341.4/501.8	51.1	4.8	371.0
2007	50.8/74.0	12.6/13.6	315.4/491.3	*n*/*a* ^2^	*n*/*a* ^2^	*n/a* ^ 2^
2008	61.9/101.6	8.8/10.9	428.9/728.2	*n*/*a* ^2^	*n*/*a* ^2^	*n/a* ^ 2^
2009	52.8/91.4	7.2/7.2	368.3/673.3	*n*/*a* ^2^	*n*/*a* ^2^	*n/a* ^ 2^

^1^All death certificates have been independently reviewed by two clinicians. We report two rates, where prostate cancer has been classified as a definite cause of death/and combined definite or probable cause of death.

^2^Mortality data for 2007 to 2009 not available (*n*/*a*).
